# MOF Template-Derived Carbon Shell-Embedded CoP Hierarchical Nanosheet as Bifunctional Catalyst for Overall Water Splitting

**DOI:** 10.3390/nano13172421

**Published:** 2023-08-25

**Authors:** Mei-Jun Liu, Fu-Hao Yang, Ji-Cheng Mei, Xu Guo, Hua-Yang Wang, Meng-Yao He, Yu-Ang Yao, Hai-Feng Zhang, Cheng-Bin Liu

**Affiliations:** 1 School of Chemical Engineering, Northeast Electric Power University, Jilin 132012, China; 20203003@neepu.edu.cn (M.-J.L.); 2202200810@neepu.edu.cn (F.-H.Y.); meijicheng@hnu.edu.cn (J.-C.M.); 2021304040115@neepu.edu.cn (X.G.); 2021304040226@neepu.edu.cn (H.-Y.W.); 2202100766@neepu.edu.cn (M.-Y.H.); 2202200809@neepu.edu.cn (Y.-A.Y.); 2 State Key Laboratory of Chemo/Biosensing and Chemometrics, College of Chemistry and Chemical Engineering, Hunan University, Changsha 410082, China

**Keywords:** transition metal phosphide, metal–organic framework, hierarchical nanosheet, overall water splitting

## Abstract

The design of earth-abundant and highly efficient bifunctional electrocatalysts for hydrogen evolution and oxygen evolution reactions (HER/OER) is crucial for hydrogen production through overall water splitting. Herein, we report a novel nanostructure consisting of vertically oriented CoP hierarchical nanosheet arrays with in situ-assembled carbon skeletons on a Ti foil electrode. The novel Zeolitic Imidazolate Framework-67 (ZIF-67) template-derived hierarchical nanosheet architecture effectively improved electrical conductivity, facilitated electrolyte transport, and increased the exposure of the active sites. The obtained bifunctional hybrid exhibited a low overpotential of 72 mV at 10 mA cm^−2^ and a small Tafel slope of 65 mV dec^−1^ for HER, and an improved overpotential of 329 mV and a Tafel slope of 107 mV dec^−1^ for OER. Furthermore, the assembled C@CoP||C@CoP electrolyzer showed excellent overall water splitting performance (1.63 V) at a current density of 10 mA cm^−2^ and superior durability. This work provides a structure engineering strategy for metal–organic framework (MOF) template-derived hybrids with outstanding electrocatalytic performance.

## 1. Introduction

Hydrogen (H_2_), with a higher gravimetric energy density than other fuels, can be produced from water alone, making it the primary choice for future energy production [1]. As a clean, renewable, and efficient method for hydrogen production, electrocatalytic water splitting has attracted extensive attention [1]. Because hydrogen evolution reaction (HER) and oxygen evolution reaction (OER) electrocatalysts usually function optimally under acidic and alkaline conditions, respectively, these electrodes often suffer from incompatibility during the coupling process. Additionally, OER, characterized by sluggish reaction kinetics, involves multi-step electron transfer, necessitating a higher overpotential than HER to provide the same current density [2]. Therefore, the development of effective electrocatalysts for alkaline media is highly desired in an overall water splitting system. Although noble metals such as Pt, Ir, and Ru and their oxides have proven to be relatively efficient HER and OER catalysts, their high cost and limited reserves hinder their large-scale application [3,4]. Therefore, it remains a great challenge to develop non-noble metal bifunctional electrocatalysts capable of both HER and OER functions under the same alkaline conditions.

The high electrocatalytic activity, low cost, and environmental friendliness of transition metal phosphides (TMPs) make them effective catalysts; thus, they have gained extensive research interest [5,6]. Recent studies have demonstrated that TMPs are promising candidates for bifunctional electrocatalysts owing to their excellent performance for both HER and OER [7,8]. Despite the remarkable progress in recent research, the existing catalysts are still not satisfactory for practical applications. Most of the TMPs suffer from low intrinsic conductivity and the easy self-agglomeration of nanocatalysts, which lead to high overpotential and a limited reaction kinetic rate in OER. Coupling TMPs with carbon-based materials can improve their electrocatalytic activity [9], and significant efforts have been made in previous works to optimize the compatibility of the composite materials [10,11]. In addition, the durability of catalysts during long-term operation under highly concentrated alkaline solutions is essential for the electrochemical water splitting reaction. Limited durability hampers the large-scale application of many existing catalysts.

Recent studies have shown that electrocatalysts with excellent nanostructures can achieve superior electrochemical performance by being directly designed and grown on a current collector. Catalysts prepared through in situ growth methods possess various advantages, including easy electrolyte penetration, good electrical conductivity, and sufficient exposure of active sites [12]. Owing to their large surface areas, intrinsic porosity, and unique composition, metal–organic frameworks (MOFs) have been widely explored as self-sacrificing templates for self-doped carbon, effectively enhancing the performance of electrocatalysts [13]. Among the various MOFs, ZIF-67 is a self-assembled structure formed by interconnection between cobalt ions and 2-methylimidazole, which has been explored for various applications [14]. Lou et al. applied a cobalt-based MOF as a template to prepare a cobalt nanoparticle-embedded C@Co_9_S_8_ double-shell nanocage structure, which exhibited excellent electrocatalytic ORR performance [15]. Yin et al. partially converted a cobalt phosphide-based MOF into CoP, and the resulting CoP/Co-MOF composites exhibited certain HER catalytic performance across a wide pH range [16]. Inspired by the above discussion, we combined the in situ preparation method with MOF template-derived carbon to create high-performance bifunctional catalysts for overall water splitting.

In this work, a C@CoP hierarchical nanosheet array with a novel nanostructure was in situ synthesized on the surface of a Ti foil substrate. This structure was utilized as an efficient bifunctional catalyst for electrochemical total water splitting. The prepared nanostructures offer several advantages that are highly desirable for high-performance electrochemical overall water splitting catalysts: (1) The nanomaterials derived from the MOF template inherit the characteristics of their precursor, possessing a high specific surface area and porous structure. This feature significantly increases the number of active sites, thus effectively improving the efficiency of electrocatalytic overall water splitting. (2) The introduction of a carbon skeleton enables the catalyst to adopt a unique multilevel nanosheet structure. This structure effectively accelerates the interfacial transfer of electrons, electrolytes, and gas products on the electrode’s surface. (3) The in situ preparation method avoids the need for a polymer binder. This not only reduces the contact resistance between the catalyst and the electrode substrate but also enhances the structural stability of the catalyst and ensures long-term performance under strong alkaline conditions. Therefore, C@CoP presents low overpotentials of 72 and 329 mV at 10 mA cm^−2^ for HER and OER, respectively, in alkaline media. Furthermore, the assembled C@CoP||C@CoP electrolyzer shows superior overall water splitting performance (1.94 V) to Pt/C||Ir/C (2.07 V) at a current density of 100 mA cm^−2^.

## 2. Materials and Methods

### 2.1. Chemicals

Cobalt nitrate hexahydrate (Co(NO_3_)_2_·6H_2_O) and 2-methylimidazole (C_4_H_6_N_2_) were purchased from Aladdin. Sodium hypophosphite monohydrate (NaH_2_PO_2_·H_2_O) was obtained from Sinopharm Chemical Reagent Co., Ltd., China. Nafion 117 solution (5 wt%), TEA ((C_2_H_5_)_3_N), and titanium foil (Ti) were provided by Sigma-Aldrich. Commercial 20 wt% Pt/C catalyst and 20 wt% Ir/C catalyst were obtained from Premetek. All of these chemicals were used without further purification. The ultrapure water (>18.25 MΩ cm) used in all experiments was purified using a Milli Q water purification system.

### 2.2. Materials Fabrication

Synthesis of Co(OH)_2_ film on Ti foil substrate: A piece of Ti foil (50 × 10 × 1 mm^3^) was first cleaned in dilute HF concentration (~8%) and successively placed in acetone, ethanol, and deionized water, with ultrasonic treatment for 10 min to remove the surface oxide layer and oil stains. A conventional three-electrode system was adopted, with a Ti foil as the working electrode and a standard Ag/AgCl electrode and a platinum plate as the reference and counter electrodes, respectively. Co(NO_3_)_2_·6H_2_O (0.1 M) was used as the electrolysis solution for the constant potential deposition at a potential of 1.0 V vs. Ag/AgCl for 400 s under magnetic stirring. Green Co(OH)_2_ film was observed on both sides of the Ti foil with an area of 30 × 10 mm^2^. The Co(OH)_2_ film was thoroughly washed to remove the surface precursor solution and then dried at room temperature. A CHI 660C electrochemical workstation (CH Instruments, Chenhua Co., Ltd., Shanghai, China) was used throughout the electrochemical processes.

Fabrication of ZIF-67@Co(OH)_2_ film: ZIF-67 was grown on the surface of Co(OH)_2_ via an in situ crystallization strategy. A total of 10 mmol of 2-methylimidazole was dissolved in deionized water (10 mL) with 1 mL TEA, and the mixture was thoroughly mixed. TEA is a deprotonation agent that can accelerate the reaction [17]. The as-prepared Co(OH)_2_ film was immersed in the solution, which was then magnetically stirred at 20 °C for 2, 5, 10, 15, and 20 min. Subsequently, the film was washed with ethanol and deionized water over 10 times, and the resulting purple films were vacuum dried at 60 °C overnight. To comparatively investigate the influence of the deprotonation agent, Co(OH)_2_ was immersed in the 2-methylimidazole solution without TEA under the same conditions.

Fabrication of C@CoP film: An NaCl protective layer was grown on the surface of ZIF-67@Co(OH)_2_ film via the salt recrystallization method to maintain the hierarchical structure. The ZIF-67@Co(OH)_2_ film electrode was directly immersed in a beaker with supersaturated NaCl solution for 10 min. After water evaporation under an infrared lamp, NaCl crystallized uniformly and encased the electrode surface. The immersion drying process was conducted five times to create a NaCl crystal-sealed electrode. The resulting ZIF-67@Co(OH)_2_ film electrode was then annealed at 700 °C for 2 h in a tube furnace at a heating rate of 2 °C min^−1^ and then naturally cooled down to room temperature to obtain the C@Co_3_O_4_ film electrode. N_2_ was continuously fed during the reaction process and the cooling process at a flow rate of 100 sccm.

The C@CoP film was prepared through the phosphorization of the C@Co_3_O_4_ film. The NaCl crystal-sealed electrode and NaH_2_PO_2_·H_2_O powder were placed in different porcelain boats (at a distance of 5 cm), with NaH_2_PO_2_·H_2_O (1 mmol) on the upstream side of the furnace. Subsequently, the furnace temperature was increased to 700 °C at a rate of 2 °C min^−1^ and maintained for 2 h in an N_2_ atmosphere, and then, the system was allowed to naturally cool to room temperature to obtain the C@CoP film. The NaCl crystal on the electrode was thoroughly washed with water, and the resulting black film was vacuum dried overnight at 60 °C. For comparison, a ZIF-67@Co(OH)_2_ film electrode without an NaCl crystal seal was subjected to the same conditions to prepare a C@CoP film electrode.

Commercial Pt/C and Ir/C membrane electrodes were prepared as standard samples for HER and OER reactions, respectively, following a reported dip-coating method [18]. Typically, 20 mg commercial catalyst powder was ultrasonically dispersed in a solution containing 1.0 mL ethanol and 50 μL Nafion (5 wt%) for 30 min to form a homogeneous ink. Subsequently, 20 μL ink was drop-casted onto the surface of a Ti foil, which was then dried in air [19].

### 2.3. Characterization

The morphology of the samples was characterized via field emission scanning electron microscopy (Hitachi S-4800, Hitachi, Tokyo, Japan) with an accelerating voltage of 5 kV. The crystal structure and detailed information on the morphology of the samples were obtained via TEM and high-resolution TEM under an accelerating voltage of 200 kV (JEM-3010, Japan Electronics Co., LTD., Hongkong, China). High-angle annular dark field scanning transmission electron microscopy, energy-dispersive X-ray spectroscopy, and elemental mapping analysis were conducted using the Tecnai G^2^ F20 S-TWIN instrument. The crystal phase of the obtained samples was determined via PXRD spectroscopy (X’Pert PRO MPD, PANalytical, Eindhoven, The Netherlands) using a Cu-Kα radiation source (*λ* = 1.5418 Å) under a constant voltage of 40 kV with a scan step of 0.02°. The chemical states of the elements in samples were determined via XPS on a Thermo Fisher Scientific K-Alpha 1063 system with monochromatic Al-Kα radiation (1486.6 eV). High-resolution scans with a narrow region for Co 2p, C 1s, and O 1s were obtained at 20 eV pass energy, 0.1 eV steps, and a 200 ms dwell time. The BEs were calibrated with a reference to the C 1s peak at 284.6 eV. The standard deviation for the BE values was 0.1 eV. Raman spectra were recorded on a Jobin–Yvon LabRam system (Horiba Jobin Yvon, Paris, France). The mass loading of CoP into the product was evaluated via inductively coupled plasma–atomic emission spectrometry (PS-6, Baird, Milwaukee, WI, USA).

### 2.4. Catalytic Performance

All electrodeposition and electrochemical measurements were performed on a CHI 660C electrochemical workstation using a three-electrode system in 1.0 M KOH at room temperature, with Ti-based electrodes as the working electrode (working area of 2 cm^2^). For HER measurements, a graphene rod and Ag/AgCl (3 M KCl) were used as counter and reference electrodes, respectively, whereas a platinum plate was used as the counter electrode for OER measurements. All potentials presented in this work were converted into RHE according to the following equation:

*E* (RHE) = *E* (Ag/AgCl) + 0.059 × pH + 0.1976 V

Prior to the tests, the electrolyte was purged by high-purity N_2_ for at least 30 min to remove the dissolved N_2_ for the HER test, and O_2_ was continuously fed to the electrolyte for at least 30 min to achieve O_2_ saturation for the OER test. The working electrodes were conditioned via CV 30 times to obtain a stable electrochemical signal. Polarization curves were obtained via linear sweep voltammetry at a scan rate of 2 mV s^−1^. The ECSAs of the catalyst in the OER and HER reactions were assessed using *C*_dl_, which was obtained from CV curves for the non-Faradaic region at sweep rates of 10 to 100 mV s^−1^. EIS was conducted at an overpotential (*η*) of 50 mV, OER measurements were conducted at 1.65 V, and HER measurements were conducted at 0.146 V; moreover, the frequency range for EIS was 100 kHz to 0.01 Hz, with an amplitude of 5 mV. The measured EIS data were fitted to an equivalent circuit model to extract the series resistance and charge-transfer resistance. The long-term durability of HER and OER was tested via continuous CV at a scan rate of 50 mV s^−1^ from −200 to 200 mV. The stability test was conducted for 60,000 s at *η* = 10 mV. The test overpotential of HER was 72 mV, whereas the test overpotential of OER was 329 mV.

Overall water splitting: An H-type water electrolyzer was applied in a two-electrode system for the overall water splitting test. A 1.0 M KOH solution was used as an electrolyte, and two symmetric titanium foil base electrodes were used as the anode and cathode. Polarization curves were obtained at a scan rate of 5 mV s^−1^. Long-term durability was assessed through continuous CV from −200 to 200 mV at a scan rate of 50 mV s^−1^. The stability test was conducted at *η* = 10 mV for 60,000 s, and the test voltage was 1.63 V. The cathode and anode chambers were connected to trace gas flowmeters to detect the production of H_2_ and O_2_ in situ.

## 3. Results and Discussion

### 3.1. Structural Analysis of the Material

**Figure 1:**
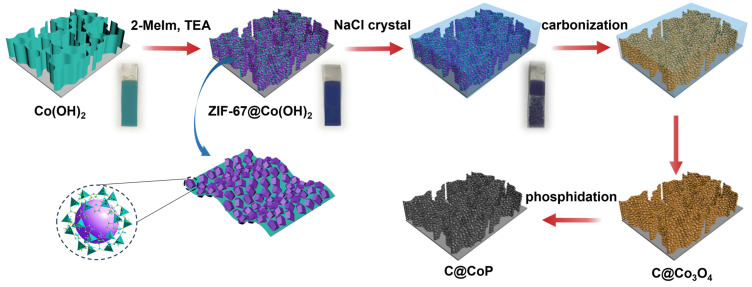
Scheme for fabrication process of C@CoP.

**Figure 2:**
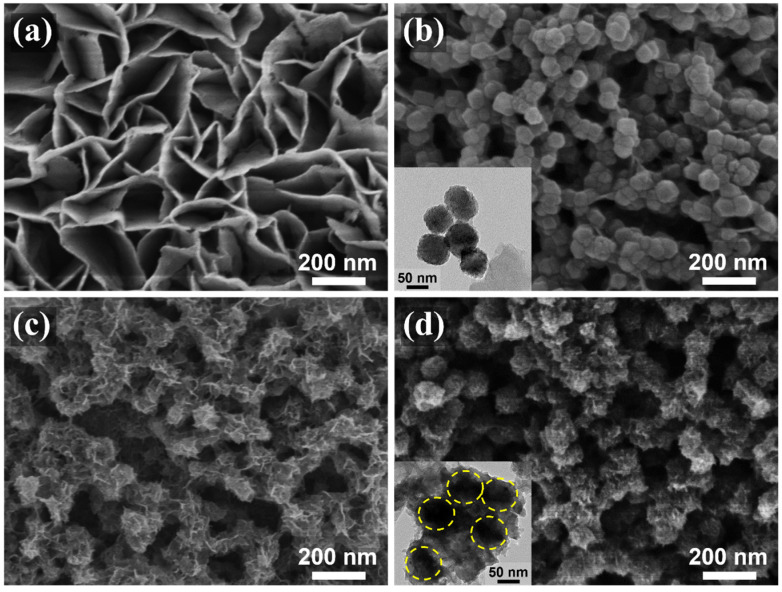
SEM images of (**a**) Co(OH)_2_, (**b**) ZIF-67@Co(OH)_2_, (**c**) C@Co_3_O_4_, and (**d**) C@CoP. The insets of (**b**,**d**) are TEM images of corresponding materials.

The fabrication of the C@CoP catalyst on the Ti foil substrate involved three main processes (Figure 1). The Co(OH)_2_ nanosheet arrays were grown on the surface of the Ti foil electrode through constant potential deposition in a CoCl_2_ electrolyte. As shown in the field emission scanning electron microscopy image (Figure 2a), the vertically oriented nanosheets were mutually interconnected, with a uniform size of several hundred nanometers. The ZIF-67 crystals were then assembled in situ on Co(OH)_2_ nanosheets in a mixture of 2-meIm and triethylamine (TEA) to form ZIF-67@Co(OH)_2_ hierarchical nanosheets (Figure 2b). The growth process of the ZIF-67 crystals under different reaction times is illustrated in Figure S1. In contrast, in the absence of TEA, ZIF-67 crystals exhibited unevenly distributed nucleation sites on the surface of Co(OH)_2_ nanosheets and increasing crystal size with increasing reaction duration. The randomly grown crystals completely blocked the structure of the Co(OH)_2_ nanosheet arrays within 5 min and subsequently detached from the Ti foil (Figure S2). In the presence of TEA, ZIF-67 crystals were uniformly deposited on 2D nanosheets, with a decreased diameter of ∼50 nm, which perfectly matched with the Co(OH)_2_ nanosheets. The separated crystals were further characterized via transmission electron microscopy (TEM, Figure 2b inset). However, ZIF-67@Co(OH)_2_-C collapsed into spherical and lump-type morphology after carbonization via traditional pyrolysis (Figure S3).

According to previous studies, the shape of carbon materials can be fixed via the salt recrystallization method [20]. After being encased in NaCl crystals, the resulting sample mostly retained the original structures of ZIF-67@Co(OH)_2_ (Figure 2c). After pyrolysis and carbonization, the ZIF-67 crystals were converted into carbon skeletons, whereas Co(OH)_2_ nanosheets and cobalt in ZIF-67 were converted into Co_3_O_4_ to form C@Co_3_O_4_ hierarchical nanosheets. The ZIF-67-derived C significantly increased the density of the electrocatalytic active sites. The surfaces of the framework were rough, with numerous carbon knots embedded in them (Figure 2c). The residual cobalt particles were embedded or encapsulated within the carbon matrix. After low-temperature phosphidation, Co_3_O_4_ was converted into CoP, and C@CoP hierarchical nanosheets were formed (Figure 2d). The TEM image shows that crystals with a diameter of ∼50 nm decorated the surface of the 2D nanosheets (Figure 2d inset). The crystal structures were more tightly coupled after annealing and inseparable from the nanosheet structure. The tightly attached crystals were beneficial to the interconnection of the conductive pathway for the electrocatalyst reactions. The produced C@CoP hierarchical nanosheets stood vertically on the surface of the Ti foil to form a highly open catalytic interface, which facilitated sufficient contact between the electrolytes and the electrode surface. The carbon skeleton, with high conductivity, was closely coupled with the nanosheets, which accelerated electron transfer at the active catalyst site.

### 3.2. Composition and Structure Characterization

**Figure 3:**
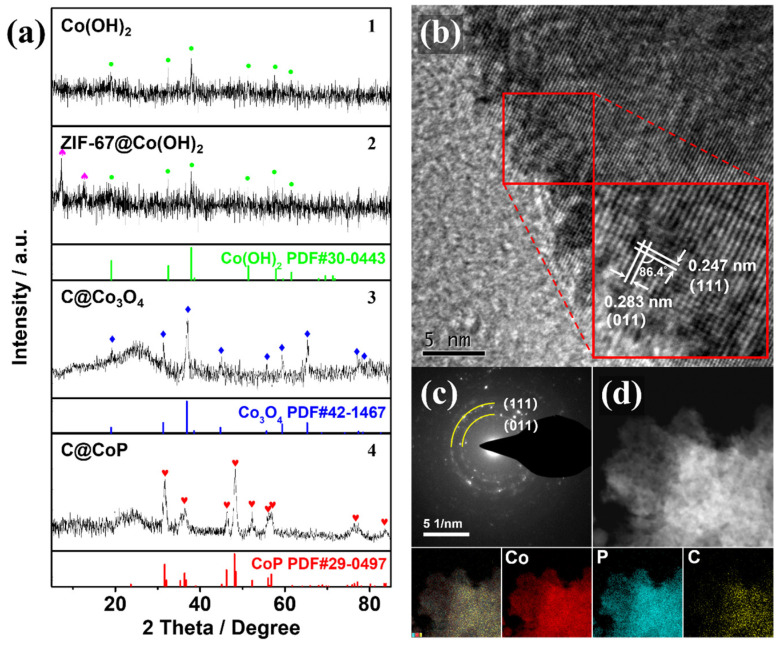
(**a**) XRD patterns of (1) Co(OH)_2_, (2) ZIF-67@Co(OH)_2_, (3) C@Co_3_O_4_, and (4) C@CoP. (**b**) HRTEM image and (**c**) SAED image of C@CoP. (**d**) STEM-EDS image and corresponding elemental mapping of C@CoP.

The SEM observation could be verified by the powder X-ray diffraction (PXRD) patterns of the powder samples at different fabrication stages. The PXRD samples collected at different stages of the fabrication process were scraped off to exclude the effect of the Ti foil substrate. The crystal structures of Co(OH)_2_, ZIF-67@Co(OH)_2_, C@Co_3_O_4_, and C@CoP are displayed in Figure 3a. The diffraction peaks of Co(OH)_2_ at 19.1°, 32.5°, 37.9°, 51.4°, 57.9°, 61.5°, 69.5°, and 71.3° corresponded to the (001), (100), (101), (102), (110), (111), (103), and (201) lattice planes of Co(OH)_2_, respectively (JCPDS card no. 30-0443) (Figure 3a1). The peaks for the electrodeposited Co(OH)_2_ film were relatively weak, indicating low crystallinity of the electrodeposited Co(OH)_2_, which is consistent with previous studies [16,20]. The overlapping peaks of ZIF-67@Co(OH)_2_ are consistent with the simulated diffraction pattern of ZIF-67, indicating that the Co(OH)_2_ nanosheets were partially reacted (Figure 3a2). The stronger diffraction peaks of Co_3_O_4_ at approximately 19.0°, 31.3°, 36.9°, 38.5°, 44.8°, 55.7°, 59.4°, 65.2°, and 77.3° could be indexed to Co_3_O_4_ (JCPDS card no. 42-1467), and the relatively weaker broad peak (2*θ* ≈ 26.2°) could be assigned to the (002) plane of graphitic carbon (Figure 3a3). The peaks from both Co(OH)_2_ and ZIF-67 were nonexistent, indicating that the Co(OH)_2_ nanosheets Co from ZIF-67 were mostly converted into pure Co_3_O_4_ after the thermal treatment under NaCl protection. The carbonization of the carbon component in the ZIF-67 crystals resulted in a highly porous structure. After phosphidation, diffraction peaks were observed at 31.6°, 36.3°, 46.2°, 48.1°, 48.4°, 52.3°, and 56.8°, consistent with the (011), (111), (112), (211), (202), (103), and (301) planes of CoP (JDPDS No. 29–0497), respectively (Figure 3a4). The results confirm the successful transformation of C@Co_3_O_4_ into C@CoP. The weaker C peak of C@CoP was a result of the lower crystallinity of samples by P doping [21].

The high-resolution TEM image (Figure 3b) confirms the crystallinity of C@CoP. The TEM image reveals lattice fringes with two identifiable *d*-spacings of 2.47 and 2.83 Å, which could be indexed to the (111) and (011) crystal planes of CoP, respectively. The dihedral angle between the two planes was measured as 86.4° [16,22]. The selected area electron diffraction (SAED) spectrum of C@CoP (Figure 3c) showed dotted rings corresponding to the crystal planes of CoP, which agrees well with the abovementioned results and reveals the polycrystalline nature of C@CoP. The spectrum featured no carbon-related diffraction ring because of the weak crystallinity. Element mapping on the structure of C@CoP was performed via scanning transmission electron microscopy coupled with energy dispersive X-ray spectroscopy (Figure 3d) to further characterize the elemental composition and distribution in the unique nanostructure. The results reveal a homogeneous distribution of the Co and P elements over the entire area, confirming the complete transformation of the Co component during the phosphidation process. Only a small amount of C element existed on the nanosheet, but C was homogeneously dispersed on the framework. These results reveal that nanoscale porous carbon was embedded into micron-scale nanosheets to form a hierarchical nanosheet architecture.

**Figure 4:**
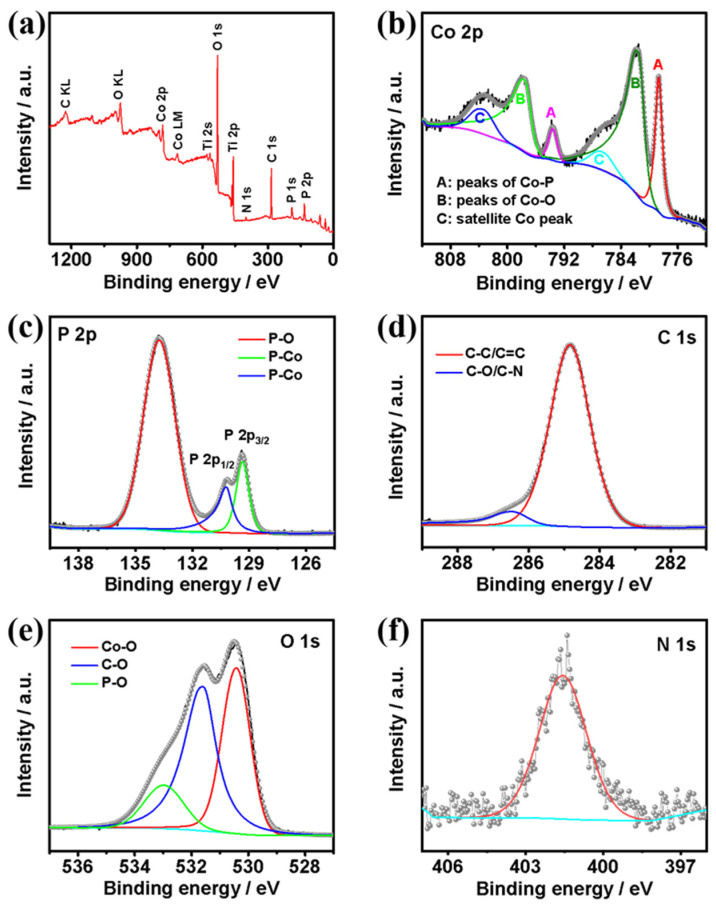
(**a**) Survey XPS spectra of C@CoP; high-resolution XPS spectra of (**b**) Co 2p, (**c**) P 2p, (**d**) C 1s, (**e**) O 1s, and (**f**) N 1 s.

X-ray photoelectron spectroscopy (XPS) measurements were further conducted to confirm the elemental composition, chemical valence states, and electron interactions of the elements on the surface of the catalyst (Figure 4). The survey spectrum (Figure 4a) reveals that the C@CoP surface was composed of Co, P, C, O, and N. The high-resolution XPS spectrum of the Co 2p spectra (Figure 4b) could be deconvoluted into six peaks. The peaks at 778.7 eV (2p_3/2_) and 793.6 eV (2p_1/2_) corresponded to the binding energies (BEs) of Co–P in CoP, whereas the apparent satellite peaks at 781.6 (2p_3/2_) and 797.6 eV (2p_1/2_) corresponded to the oxidized Co species. The intense satellite peaks located at 786.5 eV and 803.4 eV in the Co 2p spectrum corresponded to the shake-up excitation of the high-spin Co^2+^ ions in the hybrid nanosheets [23,24,25]. As shown in the P 2p spectrum (Figure 4c), the peaks at 130.5 and 129.6 eV corresponded to the Co–P bond in P 2p_1/2_ and P 2p_3/2_, respectively. The peak at 134.3 eV corresponded to P–O originating from air exposure [26]. Moreover, the BE of Co 2p_3/2_ at 778.7 eV was shifted to a higher level than that of metallic Co (777.9 eV) [27], whereas the BE of P 2p_3/2_ at 129.6 eV was shifted to a lower level than that of element P (130.2 eV) [28]. The shifts suggest that the Co carried a partial positive charge (δ+), whereas P carried a partial negative charge (δ−). Co acted as the hydride acceptor, whereas P acted as the proton acceptor, which facilitated HER [29]. The main peak centered at 284.8 eV in the C 1s spectrum (Figure 4d) corresponded to graphitic sp^2^ carbon, and the additional components centered at 286.5 eV corresponded to C–O [30,31]. Additionally, the O 1s spectrum featured three characteristic peaks at 530.3, 531.6, and 533.1 eV, corresponding to Co–O, C–O, and P–O, respectively (Figure 4e). Different from the N 1s XPS spectrum of ZIF-67@Co(OH)_2_ and C@Co_3_O_4_, the spectrum of the precursors featured a major N 1s peak. The pyridinic N was the dominant type of N species in both ZIF-67@Co(OH)_2_ (399.1 eV) and C@Co_3_O_4_ (400.4 eV). However, in the N 1s spectrum of C@CoP (Figure 4f), the peak shifted to a higher BE at 401.4 eV, corresponding to the graphitic N. This indicates that the N element in ZIF-67 likely transformed into active graphitic N sites embedded in the carbon. The doped graphitic N with stronger electronegativity led to a nonuniform electron distribution, which enabled O_2_ adsorption onto adjacent carbon atoms and benefited the catalysis reaction [32].

### 3.3. Electrocatalytic Performance

#### 3.3.1. Electrocatalytic Hydrogen Evolution

**Figure 5:**
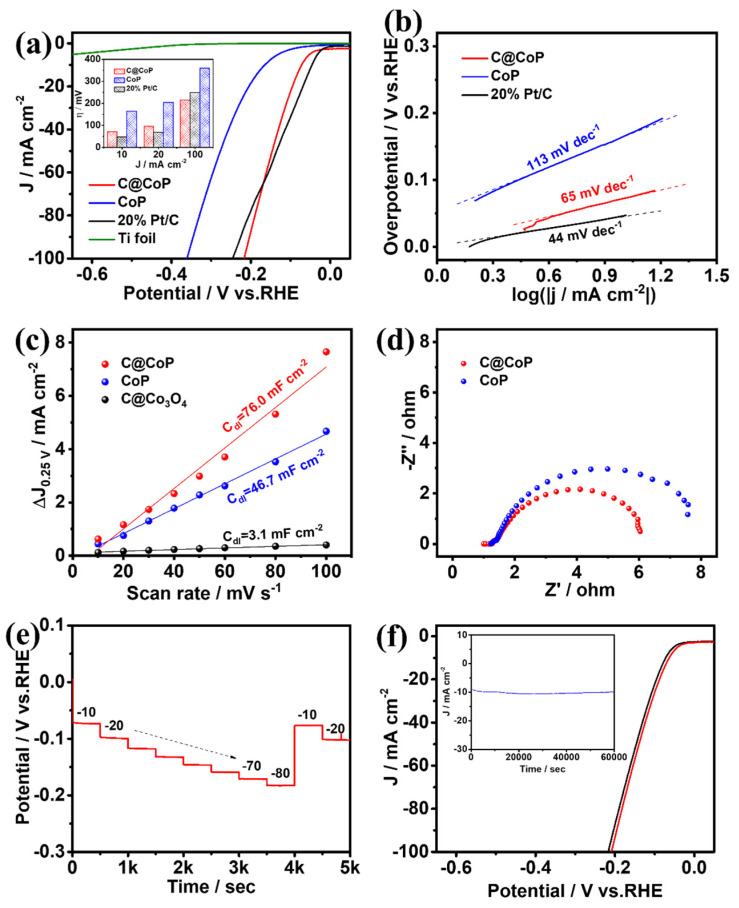
(**a**) HER polarization curves (inset shows a comparison of their overpotentials at different current densities) and corresponding (**b**) Tafel plots, (**c**) linear fitting curves of C_dl_, and (**d**) Nyquist plots for C@CoP and reference materials. (**e**) Chronopotentiometric responses of C@CoP at different current densities (10–80 mA cm^−2^). (**f**) LSV curves of C@CoP before and after 5000-cycle stability test (inset shows the I-t chronoamperometry current curve of C@CoP for 60,000 s at an overpotential of 72 mV).

The HER performances of C@CoP and the reference samples were first investigated using a typical three-electrode system with a scan rate of 2 mV s^−1^ in N_2_-saturated 1 M KOH (pH = 14). The mass loading of C@CoP on Ti foil was determined to be 2.8 mg cm^−2^ via inductively coupled plasma–optical emission spectrometry (ICP-OES). Commercial Pt/C (20 wt% platinum on Vulcan XC-72R), CoP, and bare Ti foil were also examined for comparison under identical conditions. Figure 5a shows the iR-compensated polarization curves of the as-synthesized samples. The current densities were normalized to the geometric surface areas of the Ti plate. The overpotentials of the samples at current densities of 10, 20, and 100 mA cm^−2^ are presented in the Figure 5a inset. C@CoP exhibited an overpotential of 72 mV at a low current density of 10 mA cm^−2^, which was slightly higher than that of commercial Pt/C (46 mV), but still outperformed CoP (164 mV) and ZIF-67@Co(OH)_2_ (220 mV) (Figure 5a and Figure S4a). The HER performances of C@CoP and other HER electrocatalysts are compared in Table S1. The bare Ti foil showed only negligible HER activity. Moreover, at a large current density of 100 mA cm^−2^, C@CoP exhibited the smallest overpotential (216 mV), which was much lower than that of the commercial Pt/C (255 mV). The N element in carbon can serve as an H^+^ capturer to form protonated amine groups and transport them to the CoP surface. The effect is more pronounced at high potentials, where the dynamics behavior is the key factor influencing catalytic activity [33]. The introduction of a carbon nanostructure in C@CoP considerably improved the HER activity of C@CoP, confirming that the presence of a synergistic effect between carbon and CoP led to higher catalytic activity. To further elucidate the kinetics and mechanism of the HER, Tafel slopes were derived from the corresponding polarization curves using the Tafel equation. The Tafel slope for C@CoP was estimated to be 65 mV dec^−1^, which was close to that of commercial Pt/C (44 mV dec^−1^) and much lower than that of CoP (113 mV dec^−1^) (Figure 5b and Figure S4b). The Tafel slope for C@CoP indicates that the HER proceeded via a Volmer–Heyrovsky mechanism, in which the electrochemical adsorption of H_2_O molecules (Volmer reaction) is the rate-limiting process [34]. Through extrapolation of the Tafel plot, the exchange current density for C@CoP was calculated to be 0.91 mA cm^−2^, which was quite close to that of Pt/C (0.97 mA cm^−2^). This reflects a high intrinsic rate of the electrochemical reaction on CoP/C [35]. The C@CoP catalyst exhibited a higher HER catalytic performance than most of the recently reported Co-based electrocatalysts, making it one of the best reported HER catalysts in an alkaline solution. The good catalytic activity of C@CoP is attributable to its large active area and excellent conductivity.

To further elucidate the intrinsic electrocatalytic activity of the as-prepared catalysts toward HER, the electrochemical double-layer capacitance (*C*_dl_) was measured to estimate the electrochemically active surface areas (ECSAs) [36,37]. The cyclic voltammetry (CV) curves were obtained in the non-Faradaic region of 0.2–0.3 V vs. a reversible hydrogen electrode (RHE) at different scan rates (Figure S5). The half-differences between positive and negative current densities at 0.25 V were linearly correlated with the scan rates, and the slopes of the fitting curves were considered the *C*_dl_ (Figure 5c). The calculated *C*_dl_ value for C@CoP (76.0 mF cm^–2^) was higher than that of C@Co_3_O_4_ (46.7 mF cm^–2^) and 25 times that of CoP (3.1 mF cm^–2^), suggesting that the larger ECSA was mainly due to the introduction of the ZIF-67-derived carbon framework. The higher *C*_dl_ of C@CoP was due to the highly open hierarchical structure, which provides ample accessible channels for reaction and maximal exposure of catalytic active sites; this is enhanced by the synergistic interaction between CoP and the carbon framework [38]. Electrochemical impedance spectroscopy (EIS) was conducted at an overpotential of 180 mV vs. RHE from 100 kHz to 0.01 Hz to investigate the charge and mass transportation properties (Figure 5d). The fitted lines of Nyquist plots and electrochemical impedance parameters (Table S2) were determined using an equivalent circuit model (Figure S6). In Figure S6, *R*_s_ represents the series resistance from the electrolyte and contacts. CPE and *R*_1_ represent the constant-phase element and resistance from the electron transport on the Ti foil–electrolyte interface, respectively; *C*_dl_ denotes the double-layer capacitance; and *R*_ct_ denotes the charge transfer resistance [39]. The *R*_s_ of the samples were almost similar because they were tested on the same substrate electrode and in the same testing environment. The C@CoP electrode exhibited an *R*_ct_ value of 4.30 Ω, lower than that of CoP (5.43 Ω) and much lower than that of C@Co_3_O_4_ (22.40 Ω), indicating a higher electron transport rate and thus electrical conductivity of C@CoP.

Figure 5e presents the multi-current-step chronopotentionmetric curve of C@CoP at current densities of 10 to 80 mA cm^−2^. With increasing current density, the potential increased accordingly and remained constant for the following 500 s. As the current density was reduced back to 10 and 20 mA cm^−2^, the voltage was also restored and rapidly stabilized, indicating the excellent mass transfer property of the C@CoP electrode [40]. Durability and stability are also crucial criteria for evaluating the practical utility of materials and devices. The long-term durability of C@CoP was tested by performing continuous CV cycles between −200 and 50 mV (vs. RHE) at a scan rate of 50 mV s^−1^ in a 1 M KOH solution. The linear sweep voltammetry curves of C@CoP before and after 5000 CV cycles were measured. Figure 5f shows that the overpotentials for C@CoP were well maintained, with only a negligible loss of 9 mV at −10 mA cm^−2^ after 5000 CV sweeps, demonstrating its superior HER stability. The stability of C@CoP was further evaluated via chronoamperometry tests conducted during electrolysis under a fixed overpotential of 72 mV, and no significant decline in current density occurred in a period of 60,000 s (Figure 4f inset). The morphology of C@CoP did not show a significant change after the long-term stability test (Figure S7a). However, the characteristic peak of the O 1s spectrum at 530.3 eV disappeared, which is related to the reduction of Co–O (Figure S7b). These results demonstrate that the as-prepared C@CoP material is a promising HER electrocatalyst for practical applications in the alkaline medium.

#### 3.3.2. Electrocatalytic Oxygen Evolution

**Figure 6:**
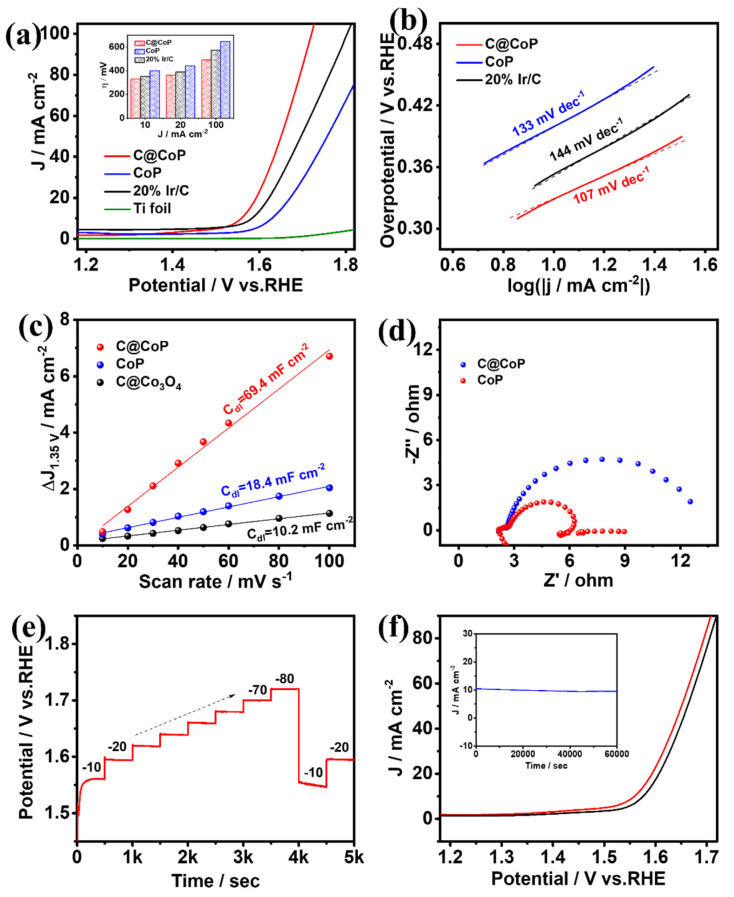
(**a**) OER polarization curves (insets show a comparison of their overpotentials at different current densities) and corresponding (**b**) Tafel plots, (**c**) linear fitting curves of C_dl_, and (**d**) Nyquist plots for C@CoP and reference materials. (**e**) Chronopotentiometric responses of C@CoP at different current densities (10–80 mA cm^−2^). (**f**) LSV curves of C@CoP before and after 5000-cycle stability test (inset shows the *I*–*t* chronoamperometry current curve of C@CoP for 60,000 s at an overpotential of 329 mV).

The same three-electrode system was applied to test the OER performances of C@CoP, commercial Ir/C (20 wt% iridium on Vulcan XC-72R), and the bare Ti foil electrode [41]. The O_2_-saturated 1.0 M KOH solution was used as the electrolyte, and the polarization curves of the samples after iR compensation were measured (Figure 6a and Figure S8a). The anode current generated by the bare Ti foil could be neglected, and the electrochemical performance was mainly determined by the electrocatalytic material loaded onto the electrode. C@CoP exhibited an overpotential of 329 mV at a current density of 10 mA cm^−2^, which was significantly better (i.e., lower) than those of commercial Ir/C (353 mV), CoP (399 mV), and ZIF-67@Co(OH)_2_ (514 mV). The OER performances of C@CoP and other reported electrocatalysts are compared in Table S3. The overpotential of C@CoP was lower than that of commercial Ir/C under various current density conditions (Figure 6a inset). The ZIF-67-induced carbon skeleton structure greatly improved the OER performance of C@CoP. The OER mechanism was investigated using the calculated Tafel slopes (Figure 6b and Figure S8b). The Tafel slope of C@CoP was 107 mV dec^−1^, which was considerably lower than those of the commercial Ir/C (144 mV dec^−1^) and CoP (133 mV dec^−1^), demonstrating that C@CoP exhibited the best OER kinetics.

The intrinsic electrocatalytic activity of the catalysts toward OER was also evaluated using the ECSA [42]. The non-Faradaic region for OER was selected as 1.3–1.4 V vs. RHE, within which CV was performed at different scanning rates (Figure S9). Following a similar method for HER, the *C*_dl_ was obtained from the fitting curves at 1.35 V. The calculated *C*_dl_ value of C@CoP was 69.4 mFcm^−2^, which was approximately three times that of C@Co_3_O_4_ (18.4 mF cm^−2^) and seven times that of CoP (10.2 mF cm^−2^) (Figure 6c). C@CoP having the highest ECSA value among the electrocatalysts is mainly related to hierarchical nanosheet architecture, which can effectively promote its OER activity. EIS was conducted at an overpotential of 420 mV vs. RHE in the frequency range of 100 kHz to 0.01 Hz (Figure 6d). The equivalent circuit model obtained from Nyquist plot fitting was the same as that of HER (Figure S6). When the same substrate electrode and testing conditions were applied, the *R*_s_ values of the various samples were almost the same (Table S4). The *R*_ct_ of the C@CoP electrode was 0.59 Ω, which was much lower than that of CoP (3.81 Ω). The higher electron transport rate indicates a higher electrical conductivity of C@CoP and thus boosted OER performance. Consistent with the HER test results, the good OER activity of C@CoP was mainly due to the combined effect of a large active area and excellent conductivity.

A multi-current-step chronopotentiometric curve was measured for the C@CoP catalyst at current densities of 10 to 80 mA cm^−2^ (Figure 6e). The potential increased with the current density and remained constant for 500 s. When the current density dropped to 10 and 20 mA cm^−2^, the voltage quickly returned to stability, indicating that the C@CoP electrode also exhibited superior mass transfer performance in the OER reaction [40]. The durability and stability of C@CoP were tested via continuous CV cycling and chronoamperometry methods, respectively. Continuous CV scans were performed in a 1 M KOH electrolyte solution at a scan rate of 50 mV s^−1^. Figure 6f shows that the polarization curve of the CoP@C electrode was not much different after 5000 CV cycles of scanning, and the overpotential at a current density of −10 mA cm^−2^ exhibited only a small loss of 17 mV, indicating excellent OER durability. A chronoamperometry test was performed at a fixed overpotential of 329 mV, and no significant decrease in current density occurred after 60,000 s (Figure 6f inset). Both the morphology and O 1s HRXPS of C@CoP showed no significant change after the long-term electrochemical test (Figure S10). The results show that the prepared CoP@C electrocatalyst also featured excellent electrocatalytic OER performance in an alkaline medium.

#### 3.3.3. Overall Water Splitting

**Figure 7:**
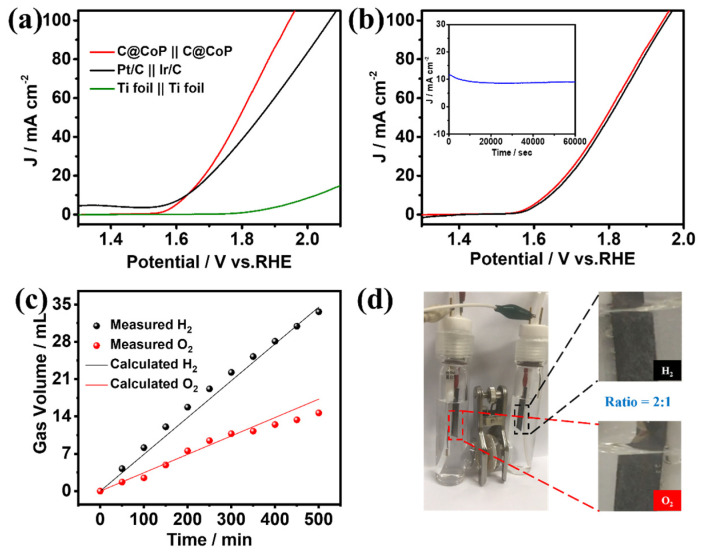
(**a**) Polarization curves of overall water splitting in 1 M KOH for C@CoP||C@CoP and other reference assembled cells. (**b**) Polarization curves of C@CoP||C@CoP before and after overall water splitting (inset shows the *I*–*t* chronoamperometry current curve of C@CoP||C@CoP for the 60,000 s stability test. (**c**) The measured and calculated output of H_2_ and O_2_ under a current density of 10 mA cm^−2^ over time. (**d**) demonstration of an actual water splitting device.

The superior HER and OER performances of C@CoP render it a suitable bifunctional catalyst for overall water splitting. A two-electrode alkaline setup was assembled using a C@CoP film loaded on a Ti foil as both the anode and cathode. C@CoP||C@CoP required a low cell voltage of only 1.63 V to deliver a current density of 10 mA cm^−2^ (Figure 7a), similar to the requirement of Pt/C||Ir/C under the same condition. Furthermore, the C@CoP||C@CoP electrolyzer achieved a high current density of 100 mA cm^−2^ at a cell voltage of 1.94 V, which was considerably lower than that of Pt/C||Ir/C (2.07 V) and most previously reported electrocatalysts (Table S5). The remarkable stability of C@CoP||C@CoP was further confirmed via a continuous *I*–*t* test at a current density of 10 mA cm^−2^ for 60,000 s, with only slight attenuation observed (Figure 7b inset). The polarization curves of C@CoP||C@CoP before and after overall water splitting were further compared, and the overpotential at a current density of 10 mA cm^−2^ showed only a small loss of 14 mV (Figure 7b). The gas volumes produced over time on the cathode and anode during the water splitting were recorded (Figure 7c). The calculated results show that the gas yields of H_2_ and O_2_ were 69.6 and 34.8 µL min^−1^, respectively, which conformed to a ratio of 2:1 (Figure 7d). This result is consistent with the theoretical values, and the Faradaic efficiencies of both HER and OER were close to 100%. The excellent electrocatalytic activity and high stability of C@CoP were due to its unique architecture. The vertical array structure of C@CoP provides a highly open space, which is beneficial for the rapid transmission of ions and generated gases. Moreover, the hierarchical nanosheet offers abundant active sites for HER and OER and facilitates electron transport through the strong interaction between the CoP and the carbon skeleton. Overall, the results confirm the potential of C@CoP as a low-cost and efficient bifunctional electrocatalyst for overall water splitting.

## 4. Conclusions

We report a novel bifunctional electrocatalyst C@CoP hierarchical nanosheet composed of vertical CoP- and ZIF-67-induced carbon skeletons. The multilevel structure was fabricated through one-step electrodeposition followed by the in situ assembly of ZIF-67 crystals. The nanostructure was well preserved after carbonization and low-temperature phosphidation under the protection of NaCl crystals. The resulting binder-free bifunctional electrocatalyst exhibited excellent performance for both HER and OER, and the assembled C@CoP||C@CoP electrolyzer showed superior overall water splitting performance, with a Faradaic efficiency of close to 100%. The outstanding catalytic performance is attributable to the vertical hierarchical nanosheet array architectures. The introduction of the carbon skeleton effectively improved electrical conductivity, facilitated electrolyte transport, and increased the exposure of the active sites. Moreover, this study demonstrated the feasibility of combining the in situ preparation method with MOF template-derived carbon to construct a hierarchical nanosheet catalyst for electrochemical overall water splitting. Replacing ZIF-67 with the MOF species of other corresponding metals allows for the potential extension of the proposed method for the preparation of diverse TMP-based energy storage and conversion electrocatalysts.

## Data Availability

The data presented in this study are available on request from the corresponding author.

## Supplementary Materials

The following supporting information can be downloaded at: https://www.mdpi.com/article/10.3390/nano13172421/s1, Figure S1: SEM images of ZIF-67 crystals grown on Co(OH)_2_ nanosheets for (a) 2 min, (b) 5 min, (c) 10 min, and (d) 15 min; Figure S2: SEM images of ZIF-67 crystals grown on Co(OH)_2_ nanosheets for (a) 2 min and (b) 5 min without the acceleration of TEA; Figure S3: SEM image of ZIF-67@Co(OH)_2_-C prepared by directly pyrolytic carbonization of ZIF-67@Co(OH)_2_; Figure S4: (a) HER polarization curves and corresponding (b) Tafel plots for C@CoP and reference materials; Figure S5: CV curves of (a) C@Co_3_O_4_, (b) CoP, and (c) C@CoP in the range of 0.2–0.3 V vs. RHE. The curves from inside to outside correspond to scanning rates of 10, 20, 30, 40, 50, 60, 80, and 100 mV s^−1^, respectively; Figure S6: Equivalent circuit fitted according to the Nyquist diagram in Figure 5d and Figure 6d; Figure S7: (a) SEM image and (b) high-resolution XPS spectra of O 1s of C@CoP after long-term HER test; Figure S8: (a) OER polarization curves and corresponding (b) Tafel plots for C@CoP and reference materials; Figure S9: CV curves of (a) C@Co_3_O_4_, (b) CoP, and (c) C@CoP in the range of 1.3–1.4 V vs. RHE. The curves from inside to outside correspond to scanning rates of 10, 20, 30, 40, 50, 60, 80, and 100 mV s^−1^, respectively; Figure S10: (a) SEM image and (b) high-resolution XPS spectra of O 1s of C@CoP after long-term OER test; Table S1: Comparison of HER performance for C@CoP with other HER electrocatalysts; Table S2: Electrochemical impedance parameters obtained by fitting the Nyquist plots of Figure 5d to the equivalent circuit model; Table S3: Comparison of OER performance of C@CoP with other reported electrocatalysts; Table S4: Electrochemical impedance parameters obtained by fitting the Nyquist plots of Figure 6d to the equivalent circuit model; Table S5: Comparison of overall water splitting performance of C@CoP with recent representative works. References [43,44,45,46,47,48,49,50,51,52,53,54] are cited in the supplementary materials.
