# Supplementary material for: MOF Template-Derived Carbon Shell-Embedded CoP Hierarchical Nanosheet as Bifunctional Catalyst for Overall Water Splitting
Source: Nanomaterials (Basel). 2023 Aug 25;13(17):2421. doi: 10.3390/nano13172421 (PMC10489850; doi:10.3390/nano13172421)
Supplement: Supplementary file 1 [file nanomaterials-13-02421-s001.zip › nanomaterials-2545690-supplementary.pdf]

Supporting Information

MOF Template-Derived Carbon Shell Embedded CoP Hierarchical Nanosheet as Bifunctional Catalyst for Overall Water Splitting

Meijun Liu[a,b], Fuhao Yang[a], Jicheng Mei[a,b], Xu Guo[a], Huayang Wang[a], Mengyao He[a], Yuang Yao[a], Haifeng Zhang[a,*], Chengbin Liu[b,*]

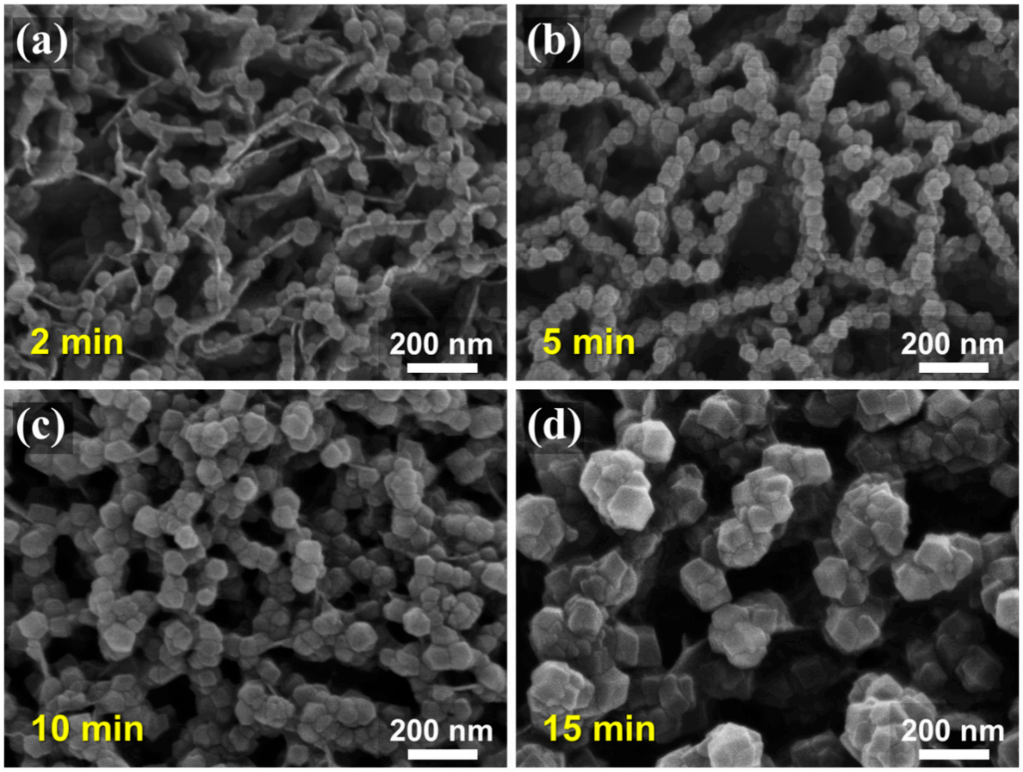

Figure S1. SEM images of ZIF-67 crystals grown on $Co(OH)_2$ nanosheets for (a) 2 min, (b) 5 min, (c) 10 min and (d) 15 min.

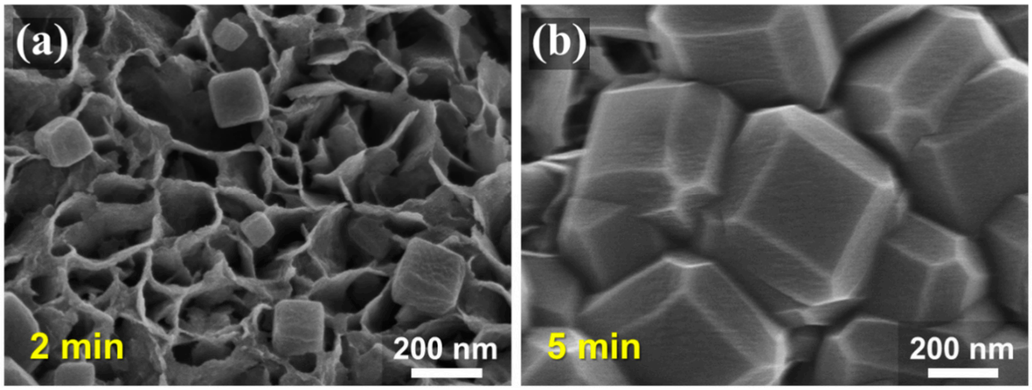

Figure S2. SEM images of ZIF-67 crystals grown on $Co(OH)_2$ nanosheets for (a) 2 min and (b) 5 min without the acceleration of TEA.

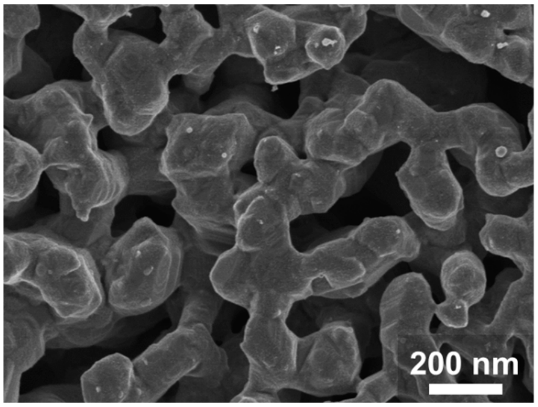

Figure S3. SEM image of ZIF-67@Co(OH)2-C prepared by directly pyrolytic carbonization of ZIF-67@Co(OH)2.

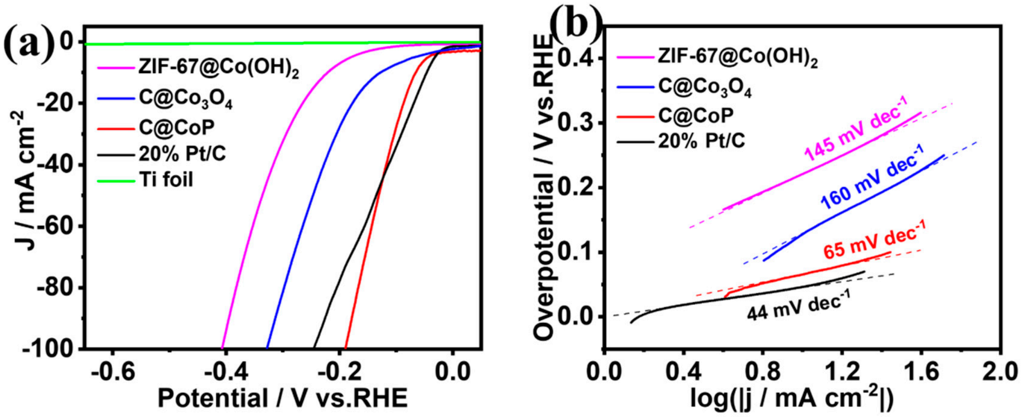

Figure S4. (a) HER Polarization curves and corresponding (b) Tafel plots for C@CoP and reference materials.

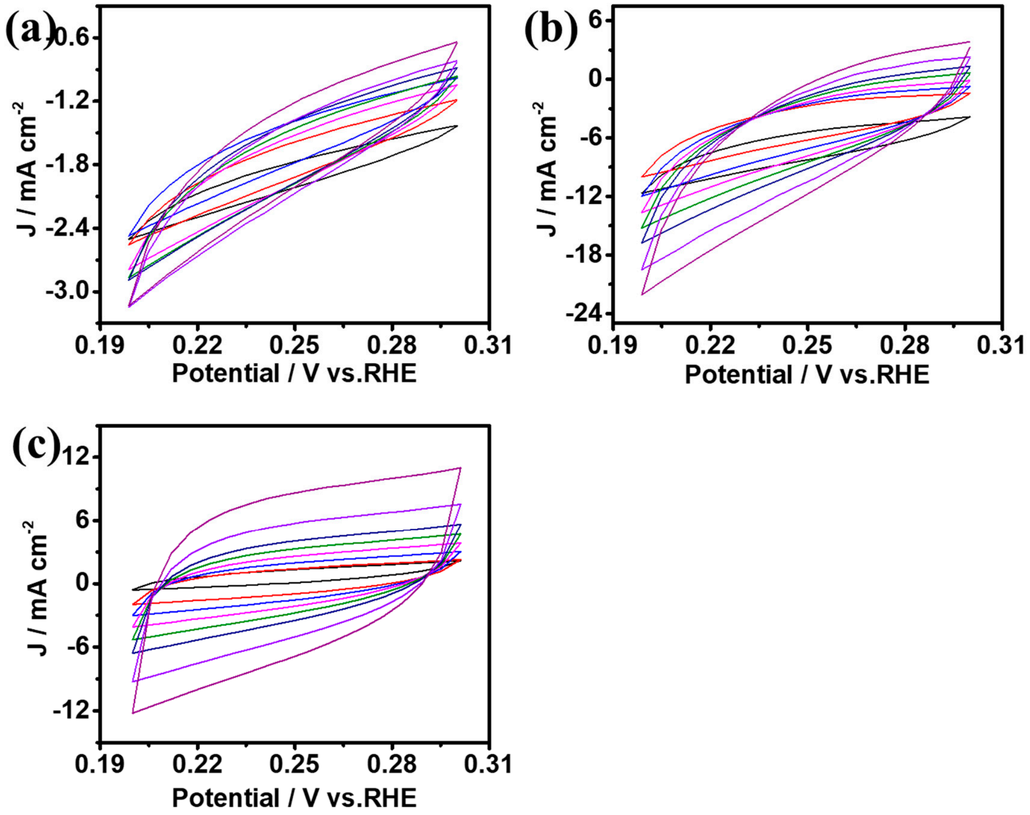

Figure S5. CV curves of (a) C@Co_3O_4, (b) CoP and (c) C@CoP in the range of 0.2−0.3 V vs.RHE. The curves from inside to outside correspond to the scanning rate of 10, 20, 30, 40, 50, 60, 80 and 100 mV s -1, respectively.

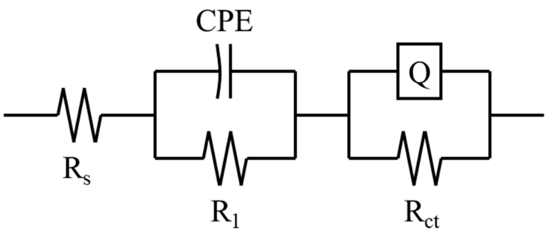

Figure S6. Equivalent circuit fitted according to the Nyquist diagram in Figure 5d and Figure 6d .

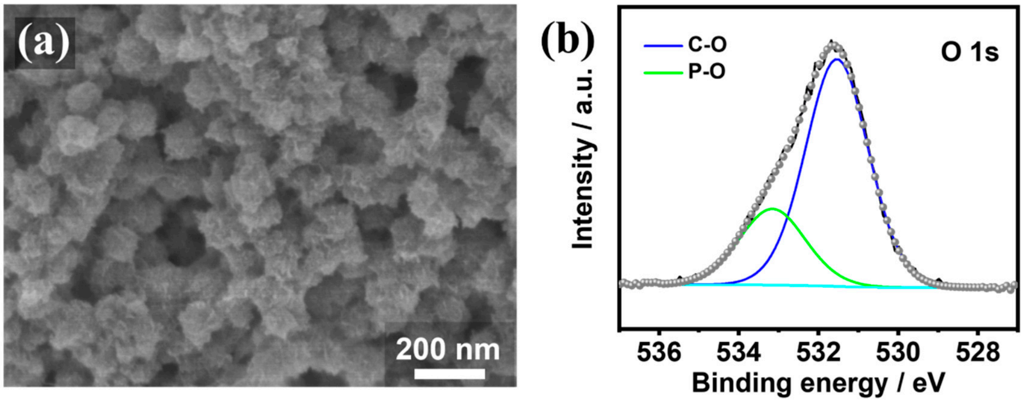

Figure S7. (a) SEM image and (b) high-resolution XPS spectra of O 1s of C@CoP after long term HER test.

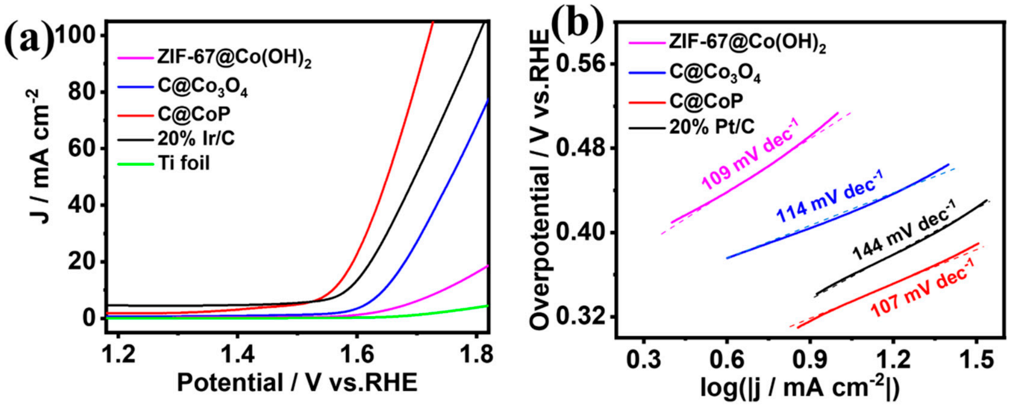

Figure S8. (a) OER Polarization curves and corresponding (b) Tafel plots for C@CoP and reference materials.

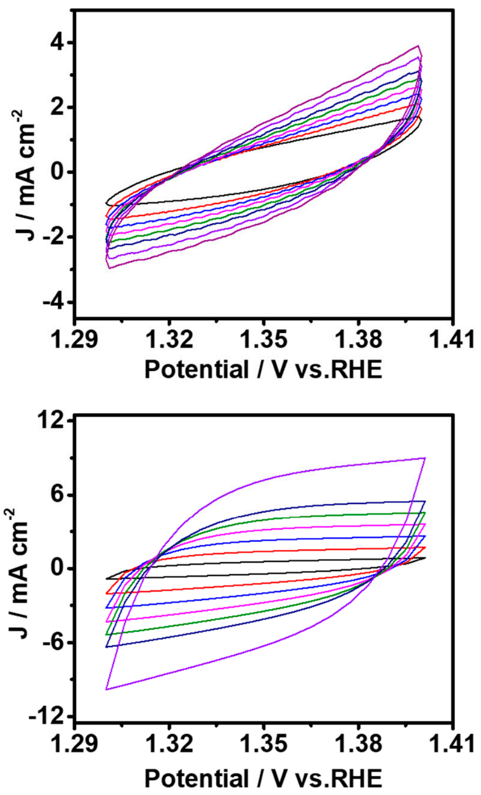

Figure S9. CV curves of (a) $C@Co_3O_4$, (b) CoP and (c) C@CoP in the range of 1.3−1.4 V vs.RHE. The curves from inside to outside correspond to the scanning rate of 10, 20, 30, 40, 50, 60, 80 and 100 mV s -1, respectively.

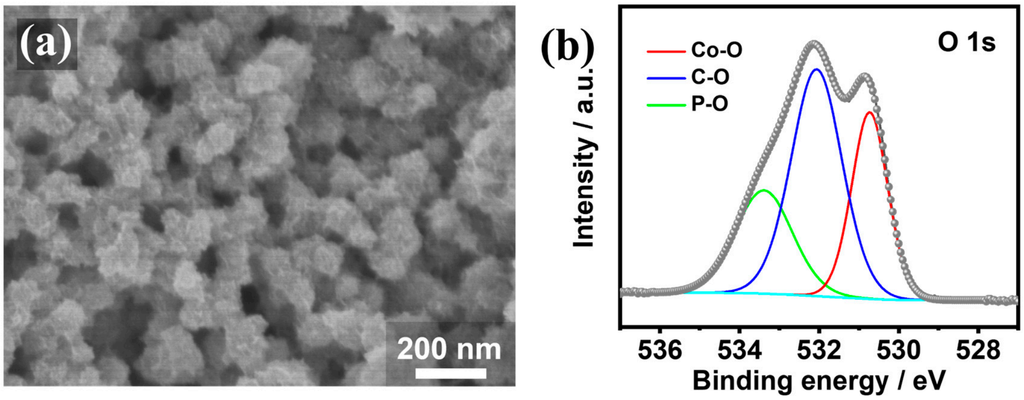

Figure S10. (a) SEM image and (b) high-resolution XPS spectra of O 1s of C@CoP after long term OER test.

Table S1. Comparison of HER performance for C@CoP with other HER electrocatalysts

Catalyst	Overpotential (mV) at 10 mA/cm^2	Tafel slope (mV/dec)	Electrolyte	Reference
**C@CoP**	72	65	1.0 M KOH	This work
**Ni-CoP/HPFs**	144<br>92	62<br>34	0.5 M H_2SO_4<br>1.0 M KOH	1
**Mn_2P-Mn_2O_3/PNCF**	98	46	1.0 M KOH	2
**FePx/ Fe-N-C /NPC**	75<br>182	60<br>132	0.5 M H_2SO_4<br>1.0 M KOH	3
**CoP/Co-MOF**	27<br>34<br>49	43<br>56<br>63	0.5 M H_2SO_4<br>1.0 M KOH<br>1.0M PBS	4
**PMA@ZIF-67-C-AT**	570	222	0.2M PBS	5
**Co_5Fe_5-C**	165	70.7	1.0 M KOH	6
**V-CoP_2/CC**	50	32	0.5 M H_2SO_4	7
**VCoCOx@NF**	63	93	1.0 M KOH	8
**Co_2P/CoP@Co@NCNT**	118<br>136	46<br>49	1.0 M KOH<br>0.5 M H_2SO_4	9

Table S2. Electrochemical impedance parameters obtained by fitting the Nyquist plots of Figure 5d to the equivalent circuit model

Catalyst	R_s (Ω)	C (mF cm^{-2})	R_1 (Ω)	Q		R_{ct} (Ω)
				$Y_1(\Omega^{-1}s^n)$	n	
C@CoP	1.01	3.89	4.30	2.56×10^{-3}	0.480	4.30
CoP	1.17	5.43	5.43	5.03×10^{-3}	0.254	5.43
C@Co_3O_4	1.22	0.966	22.40	5.98×10^{-3}	0.872	22.40

Table S3. Comparison of OER performance of C@CoP with other reported electrocatalysts.

Catalyst	Overpotential (mV) at 10 mA/cm^2	Tafel slope (mV/dec)	Electrolyte	Reference
**C@CoP**	329	107	1.0 M KOH	This work
**Co_5Fe_5-C**	245	58.2	1.0 M KOH	[6]
**$W_{0.2}Er_{0.1}Ru_{0.7}O_{2-\delta}$**	168	66.8	0.5M H_2SO_4	[10]
**np-Ir/NiFeO**	197	29.6	1.0 M KOH	[11]
**Mn_2P-Mn_2O_3/PNCF**	370	86	1.0 M KOH	[2]
**FePx/ Fe-N-C /NPC**	325	79	1.0 M KOH	[3]
**NiMoP@NiFe-LDH**	299	23.3	1.0 M KOH	[12]
**V-CoP2/CC**	91	40	0.5 M H_2SO_4	[7]
**VCoCOx@NF**	240	65	1.0 M KOH	[8]
**Co_2P/CoP@Co@NCNT**	256	46	1.0 M KOH	[9]

Table S4. Electrochemical impedance parameters obtained by fitting the Nyquist plots of Figure 6d to the equivalent circuit model

Catalyst	R_s (Ω)	C (mF cm^{-2})	R_1 (Ω)	Q		R_{ct} (Ω)
				$Y_1(\Omega^{-1}s^n)$	n	
C@CoP	2.17	4.90	9.66	6.75×10^{-3}	0.604	0.59
CoP	2.68	6.75	8.55	4.45×10^{-3}	0.968	3.81

Table S5. Comparison of overall water splitting performance of C@CoP with recent representative works.

Catalyst	Cell voltage (V) at 10 mA/cm^2	Electrolyte	Reference
**C@CoP**	1.63	1.0 M KOH	This work
**Mn_2P-Mn_2O_3/PNCF**	1.6	1.0 M KOH	[2]
**FePx/ Fe-N-C /NPC**	1.58	1.0 M KOH	[3]
**Ir_1@Co/NC**	1.603	1.0 M KOH	[13]
**Co-Fe NPs**	1.92	1.0 M KOH	[14]
**Fe-NiS_2/CF**	1.722	1.0 M KOH	[15]
**O-$Ni_{0.5}W_0$.5Se_2**	1.56	1.0 M KOH	[16]
**$Co_{0.75}Fe_{0.25}P$**	1.63	1.0 M KOH	[17]
**Co_2P/CoP@Co@NCNT**	1.6	1.0 M KOH	[9]
**Co/Mo2C@NC800-2**	1.67	1.0 M KOH	[18]

1. Pan, Y.; Sun, K.; Lin, Y.; Cao, X.; Cheng, Y.; Liu, S.; Zeng, L.; Cheong, W.-C.; Zhao, D.; Wu, K.; Liu, Z.; Liu, Y.; Wang, D.; Peng, Q.; Chen, C.; Li, Y., Electronic structure and d-band center control engineering over M-doped CoP (M = Ni, Mn, Fe) hollow polyhedron frames for boosting hydrogen production. *Nano Energy* **2019,** *56*, 411-419.
2. Wang, X.; Huang, G.; Pan, Z.; Kang, S.; Ma, S.; Shen, P. K.; Zhu, J., One-pot synthesis of Mn2P-Mn2O3 heterogeneous nanoparticles in a P, N -doped three-dimensional porous carbon framework as a highly efficient bifunctional electrocatalyst for overall water splitting. *Chemical Engineering Journal* **2022,** *428*.
3. Qin, Q.; Jang, H.; Li, P.; Yuan, B.; Liu, X.; Cho, J., A Tannic Acid-Derived N-, P-Codoped Carbon-Supported Iron-Based Nanocomposite as an Advanced Trifunctional Electrocatalyst for the Overall Water Splitting Cells and Zinc-Air Batteries. *Advanced Energy Materials* **2019,** *9* (5).
4. Liu, T.; Li, P.; Yao, N.; Cheng, G.; Chen, S.; Luo, W.; Yin, Y., CoP-Doped MOF-Based Electrocatalyst for pH-Universal Hydrogen Evolution Reaction. *Angew Chem Int Ed Engl* **2019,** *58* (14), 4679-4684.
5. Zhao, X.; Zhang, Q.; Huang, X.; Ding, L.; Yang, W.; Wang, C.; Pan, Q., Polyoxometalate@ZIF-67 derived carbon-based catalyst for efficient electrochemical overall seawater splitting and oxygen reduction. *International Journal of Hydrogen Energy* **2022,** *47* (4), 2178-2186.
6. Yan, Y.; Han, Y.; Wang, F.; Hu, Y.; Shi, Q.; Diao, G.; Chen, M., Bifunctional electrocatalyst of CoxFey-C for overall water splitting. *Journal of Alloys and Compounds* **2022,** *897*.
7. Wang, Y.; Jiao, Y.; Yan, H.; Yang, G.; Tian, C.; Wu, A.; Liu, Y.; Fu, H., Vanadium-Incorporated CoP(2) with Lattice Expansion for Highly Efficient Acidic Overall Water Splitting. *Angew Chem Int Ed Engl* **2022,** *61* (12), e202116233.
8. Meena, A.; Thangavel, P.; Nissimagoudar, A. S.; Narayan Singh, A.; Jana, A.; Sol Jeong, D.; Im, H.; Kim, K. S., Bifunctional oxovanadate doped cobalt carbonate for high-efficient overall water splitting in alkaline-anion-exchange-membrane water-electrolyzer. *Chemical Engineering Journal* **2022,** *430*.
9. Lu, Z.; Cao, Y.; Xie, J.; Hu, J.; Wang, K.; Jia, D., Construction of Co2P/CoP@Co@NCNT rich-interface to synergistically promote overall water splitting. *Chemical Engineering Journal* **2022,** *430*.
10. Hao, S.; Liu, M.; Pan, J.; Liu, X.; Tan, X.; Xu, N.; He, Y.; Lei, L.; Zhang, X., Dopants fixation of Ruthenium for boosting acidic oxygen evolution stability and activity. *Nature Communications* **2020,** *11* (1), 5368.
11. Jiang, K.; Luo, M.; Peng, M.; Yu, Y.; Lu, Y. R.; Chan, T. S.; Liu, P.; de Groot, F. M. F.; Tan, Y., Dynamic active-site generation of atomic iridium stabilized on nanoporous metal phosphides for water oxidation. *Nat Commun* **2020,** *11* (1), 2701.
12. Xiao, L.; Bao, W.; Zhang, J.; Yang, C.; Ai, T.; Li, Y.; Wei, X.; Jiang, P.; Kou, L., Interfacial interaction between NiMoP and NiFe-LDH to regulate the electronic structure toward high-efficiency electrocatalytic oxygen evolution reaction. *International Journal of Hydrogen Energy* **2022,** *47* (15), 9230-9238.
13. Lai, W.-H.; Zhang, L.-F.; Hua, W.-B.; Indris, S.; Yan, Z.-C.; Hu, Z.; Zhang, B.; Liu, Y.; Wang, L.; Liu, M.; Liu, R.; Wang, Y.-X.; Wang, J.-Z.; Hu, Z.; Liu, H.-K.; Chou, S.-L.; Dou,

S.-X., General π-Electron-Assisted Strategy for Ir, Pt, Ru, Pd, Fe, Ni Single-Atom Electrocatalysts with Bifunctional Active Sites for Highly Efficient Water Splitting. *Angewandte Chemie International Edition* **2019,** *58* (34), 11868-11873.
14. Adamson, W.; Bo, X.; Li, Y.; Suryanto, B. H. R.; Chen, X.; Zhao, C., Co-Fe binary metal oxide electrocatalyst with synergistic interface structures for efficient overall water splitting. *Catalysis Today* **2020,** *351*, 44-49.
15. Yu, C.; Huang, H.; Zhou, S.; Han, X.; Zhao, C.; Yang, J.; Li, S.; Guo, W.; An, B.; Zhao, J.; Qiu, J., An electrocatalyst with anti-oxidized capability for overall water splitting. *Nano Research* **2018,** *11* (6), 3411-3418.
16. Singh, M.; Nguyen, T. T.; Balamurugan, J.; Kim, N. H.; Lee, J. H., Rational manipulation of 3D hierarchical oxygenated nickel tungsten selenide nanosheet as the efficient bifunctional electrocatalyst for overall water splitting. *Chemical Engineering Journal* **2022,** *430*.
17. Huang, Y.; Li, M.; Yang, W.; Yu, Y.; Hao, S., 3D ordered mesoporous cobalt ferrite phosphides for overall water splitting. *Science China Materials* **2020,** *63* (2), 240-248.
18. Liu, G.; Wang, K.; Wang, L.; Wang, B.; Lin, Z.; Chen, X.; Hua, Y.; Zhu, W.; Li, H.; Xia, J., A Janus cobalt nanoparticles and molybdenum carbide decorated N-doped carbon for high-performance overall water splitting. *Journal of Colloid and Interface Science* **2021,** *583*, 614-625.
